# Radionuclide Imaging of Apoptosis in Malignancies: Promise and Pitfalls of ^99m^Tc-Hynic-rh-Annexin V Imaging

**DOI:** 10.4137/cmo.s349

**Published:** 2008-03-25

**Authors:** M.S. Kartachova, M. Verheij, B.L. van Eck, C.A. Hoefnagel, R.A. Valdes Olmos

**Affiliations:** 1 Department of Nuclear Medicine, The Netherlands Cancer Institute/Antoni van Leeuwenhoek Hospital, Amsterdam, The Netherlands; 2 Department of Radiotherapy, The Netherlands Cancer Institute/Antoni van Leeuwenhoek Hospital, Amsterdam, The Netherlands; 3 Department of Nuclear Medicine, Academic Medical Centre, University of Amsterdam, Amsterdam, The Netherlands

**Keywords:** apoptosis, ^99m^Tc-Hynic-rh-Annexin-V, imaging oncology

## Abstract

Radionuclide detection of apoptosis with of ^99m^Tc-Hynic-rh-Annexin V scintigraphy is an effective tool for in vivo visualisation and monitoring of apoptosis in various malignant tumour. Early therapy-induced increase of the tumour tracer uptake correlates with favourable outcome, whereas stable or decreased uptake correlates with stable disease or tumour progression. Therefore sequential ^99m^Tc-Hynic-rh-Annexin V scintigraphy could be used to predict therapy outcome on a patient-to-patient basis within 48 hours after the start of treatment. However, moderate tumour-to-background ratio and therapy-induced changes in normal tissues could confound image analysis. To assure accurate interpretation of Annexin V scans, the awareness of the biophysiological and biochemical properties contributing to the tracer distribution is essential. In with manuscript we discuss the patterns of Annexin V tumour uptake and illustrate the most frequent pitfalls associated with Annexin V imaging in correlation with CT and MRI imaging.

## Introduction

Radionuclide detection of apoptosis with of ^99m^Tc-Hynic-rh-Annexin V scintigraphy is an effective tool for in vivo visualisation and monitoring of apoptosis in various malignant tumours^1^,^2^. Early therapy-induced changes in tracer uptake correlate well with the therapy outcome and therefore could be used to predict therapy outcome on a patient-to-patient basis within 48 hours after the start of treatment. However, moderate tumour-to-background ratio and therapy-induced changes in normal tissues could confound image analysis. Accurate interpretation of Annexin V scans requires awareness of the biophysiological and biochemical properties contributing to the tracer distribution. Knowledge of common patterns and pitfalls associated with Annexin V imaging in combination with image corregistration (SPECT/CT) can make this modality more valuable for evaluation of responses to anticancer therapy.

## Role of Apoptosis in Oncology

Apoptosis is a key regulator of tissue homeostasis, which critically depends on the balance between the tissue proliferation and cell death compartments. Defects in apoptotic pathways are now known to contribute to a wide range of human diseases, varying from neurodegenerative disorders to malignancies^3^,^4^.

If disruption of the apoptosis process contributes to pathogenesis of cancer, then it is not surprising that existing anticancer agents work by triggering apoptosis^5^,^6^,^7^. Recent studies demonstrated that most, if not all chemotherapeutic drugs and radiotherapy induce apoptosis in susceptible cell types.

Despite extremely complicated genetic regulation of apoptotic pathways, morphological cell changes are quite predictable. The morphological hallmarks of apoptosis are cell membrane blebbing, cytoplasm shrinkage, chromatin condensation and fragmentation into membrane-closed vesicles^8^. These changes are accompanied by biochemical transformations, including externalisation of phosphatidylserine on the outer leaflet of the cell membrane^9^. This process is one of the earliest events in the apoptosis cascade. It does not depend on the apoptosis-inducing agent or cell type, persists until the cell death programme is fully completed and promotes recognition of the apoptotic cells by phagocytes. Therefore, early exposure of phosphatidylserine constitutes an attractive biomarker for non-invasive in vivo imaging of apoptosis.

## Annexin V biophysiological and biochemical properties

Annexin V is a member of the annexin family of calcium and phospholipids binding family of proteins^10^. Like the other annexin proteins, it is widely expressed in eukaryotic organisms. It consists of 319 amino acids, forming a single polypeptide chain with molecular weight of 35.7 kDA. It is folded into a planar cyclic arrangement with the NH2-terminal tail, unique for each Annexin, and COOH-protein terminal core domain, consisting of four homologous repeats of approximately 70 amino acids. Every repeat contains a highly conserved sequence of 17 amino acids termed the endonexin loop, which harbours a characteristic Ca^2+^ and phospholypid-binding site^11^.

Most of the known biological activities of Annexin V are attributed to its high affinity for 12 phosphatidylserine, it was labelled with fluorescent and radionuclide tags and is now widely used for detection and quantification of apoptosis both in vitro and in vivo.

## ^99m^Tc-Hynic-rh-Annexin V Scintigraphy

Recombinant Annexin V labelled with ^99m^Technetium by hydrazine-nicotinamide (HYNIC) method is so far the most widely investigated and extensively used tracer for in vivo visualisation of programmed cell death. It is successfully used in animal models of myocardial ischemia^13^,^14^, transplantation medicine^15^ hepatic apoptosis, inflammation and hypoxic brain injury^16^. Most interestingly it has shown great potential for in vivo monitoring of therapy-induced apoptosis in various malignant tumours as a predictor of therapy outcome^17^,^18^,^19^.

^99m^Tc-Hynic-rh-Annexin V scintigraphy is performed 4 hours after the injection of 555–925 MBq (15–25mCi) of ^99m^Tc-labeled Annexin V to allow clearance of the radiotracer from the soft tissues, resulting in a higher tumour-to-background ratio. A gamma camera equipped with low-energy, high-resolution collimator provides the best quality images. Standard acquisition includes anterior and posterior static whole body images, obtained with the matrix 1024 × 512 and SPECT of the region of interest acquired by the step-and-shoot mode, one step per 3 degrees, 30 seconds per frame, matrix size 128 × 128 × 16. For SPECT reconstructions an iterative algorithm and postfiltering using a Butterworth filter (cut off frequency 0.35, order 5) are recommended.

The time interval between the initiation of therapy and early posttreatment scan can be a critical point for correct interpretation of the tumour uptake changes. Currently posttreatment scans are performed between 4 and 48 hours after the start of treatment and depends on both the tumour morphology and treatment modality.

## General Interpretation Issues

**Figure 1:**
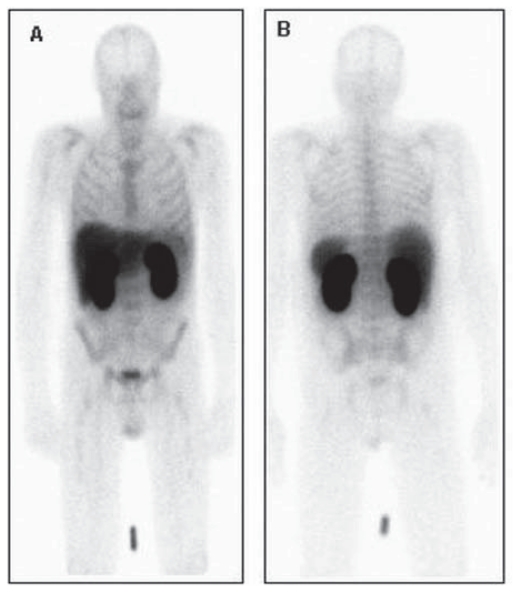
Normal distribution of ^99m^Tc-Hynic-rh-Annexin VPlanar anterior (**A**) and posterior (**B**) images, obtained 4 hours after intravenous injection of the radiofarmacon demonstrate physiological tracer accumulation in the liver, kidneys, bladder and bone marrow.

^99m^Tc-Hynic-rh-Annexin V binds selectively to phosphatidylserine (PS) residuals in the presence of Ca^2+^ ions independently of the apoptosis-inducing agent or cell type. After intravenous injection it is distributed with the bloodstream and binds to the apoptotic cells or cell debris in normal tissues and tumours. Imaging is typically started at least four hours after injection to allow sufficient blood pool clearance. Normal physiologic uptake is seen in the liver, spleen, and bone marrow. Because of predominantly renal clearance, intense accumulation is always seen in renal parenchyma, collecting system and bladder^20^. Normal distribution of ^99m^Tc-Hynic-rh-Annexin V is illustrated on Figure 1.

## Annexin V Uptake in Various Malignancies

It is widely accepted that therapy-induced changes in Annexin V tumour uptake, detected shortly after the start of anticancer treatment correlate well with therapy outcome in various malignant tumours. In our previous publications we reported the pattern of the tracer uptake in low grade lymphomas, non-small cell lung cancer (NSCLC) and head and neck squamous cell carcinoma. In general, therapy-induced increase of the tumour uptake within 48-hours after the start of treatment correlated with favourable response, stable or decreased tracer accumulation indicated stable disease or progression. Interestingly, the pattern of Annexin V tumour uptake depended to a large extent on tumour morphology and treatment regimen. Low grade lymphomas, highly radio-sensitive tumours, responding to radiotherapy predominantly through therapy-induced apoptosis^21^, served in this context a perfect model to study the feasibility of in vivo monitoring of apoptosis in malignancies. In 26 patient with low grade lymphomas we have observed moderate baseline tracer accumulation with a prominent increase of uptake in responding tumours^22^. In contrast, patients with NSCLC show low or absent uptake before the start of treatment with less prominent, however significant increase in responders^23^. The groups of Belhocine^17^ and van de Wiele^24^,^25^ had demonstrated similar pattern of tracer uptake in breast cancer, bladder and oesophageal cancer.

Thus the general pattern of therapy-induced tumour uptake changes in malignant tumours seems to be constant. Therapy-induced increase of tracer uptake correlates with favourable outcome, stable or decreased uptake—with stable disease or progression, however the intensity and grade of tracer uptake could vary depending on tumour morphology.

## Specific Interpretation Issues

### Tumour uptake

**Figure 2:**
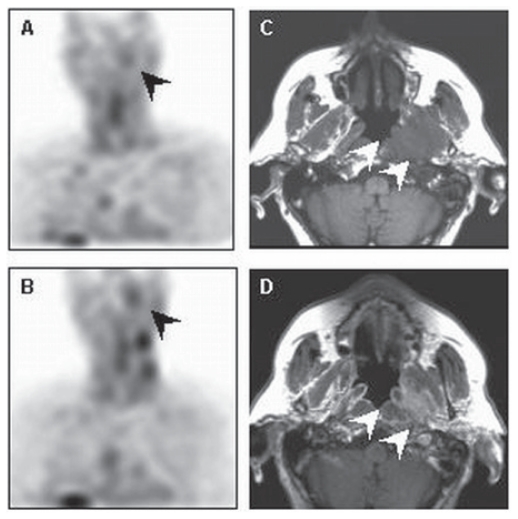
Tumour localisation A 79-year old male with low grade follicular lymphoma in lymph nodes on the left side of the neck. Coronal baseline (**A**) and early posttreatment (**B**) Annexin V SPECT images show pathological tracer accumulation in the nasopharynx left (black arrows). This lesion correlates with the solid mass on axial T1-weighted (**C**) and contrast-enhanced T1-weighted (**D**) MR images (white arrows). Histological examination revealed the diagnosis of low grade follicular lymphoma. Note radiotherapy-induced increase of the tracer uptake in the irradiated neck lymph nodes on the early posttreatment scan (**B**).

Annexin V accumulation before the start of treatment represents a combination of spontaneous apoptosis and necrosis as part of tumour cell turnover. Consequently, tracer accumulations outside the known tumour sites or areas of physiological uptake on the total body and SPECT could indicate unknown tumour localisations as illustrated on Figure 2.

Tumour uptake evaluation could be compromised by intense tracer accumulation in the surrounding normal tissues. This phenomenon is of high importance for the evaluation of the posttreatment scans, when normal tracer distribution is disturbed by therapy-induced increase of uptake in the salivary glands and bone marrow.

Introduction of such hybrid SPECT/CT systems has addressed this problem; however in absence of hybrid systems, accurate software based matching could deliver additional information about the tumour localisation and its relation with normal structures.

### Tracer uptake in benign (or non-malignant) lesions

Increased Annexin V uptake can be observed in many benign processes, including inflammation and infectious diseases (pneumonia, phlebitis), instable atherosclerotic plaques. Another possible pitfall we have observed was the tracer accumulation in capillary haemangioma.

#### Infection and inflammation

**Figure 3:**
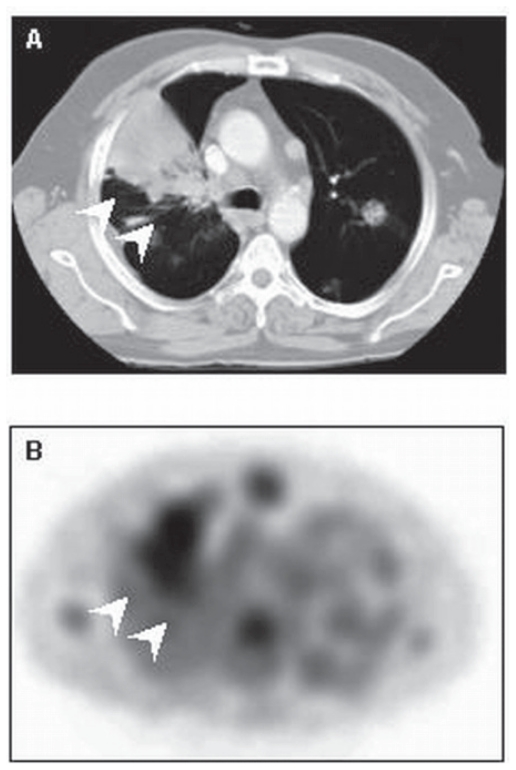
A 52-year old man with NSCLC and post-obstructive pneumonia in the right upper lobe (white arrows) on contrast-enhanced CT (**A**) which shows intense tracer uptake (white arrows) in the baseline Annexin V SPECT (**B**).

Infectious foci contain both necrotic and apoptotic cells, thus providing the substrate for Annexin V binding as illustrated in Figure 3.

#### Thrombosis and thrombophlebitis

**Figure 4:**
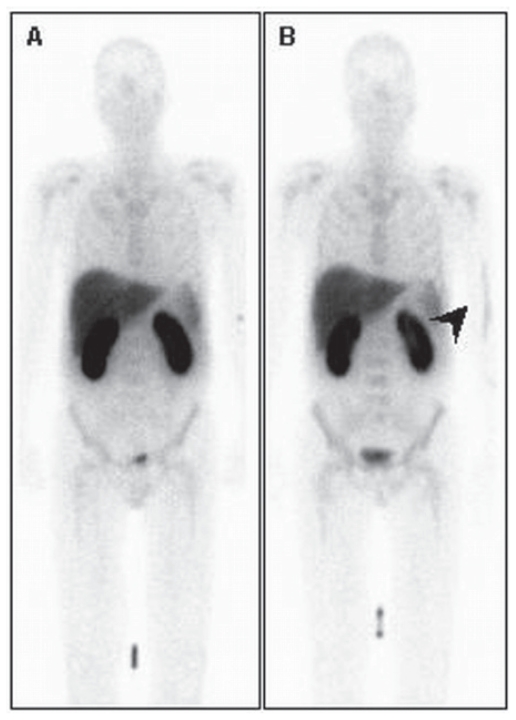
Baseline (**A**) and post-treatment (**B**) planar Annexin V images obtained 24 hours after the first injection of cisplatin in the right elbow show linear increased tracer uptake in the projection of the vena basilica, corresponding with the presence of phlebitis.

Annexin V was first introduced as a platelet-directed thrombus-targeting agent. In vivo visualisation of acute platelet-rich thrombi by means of Annexin V scintigraphy is widely discussed in the literature^26^,^27^,^28^. Tracer affinity to PS exposed on the surface of activated platelets explains tracer accumulation on the compromised vessels. This phenomenon as well as PS exposure on the inflammatory cells may be responsible for tracer accumulation at sites of thrombophlebitis after the intravenous infusion of cisplatin en gemcitabine as illustrated in Figure 4.

#### Atherosclerosis

**Figure 5:**
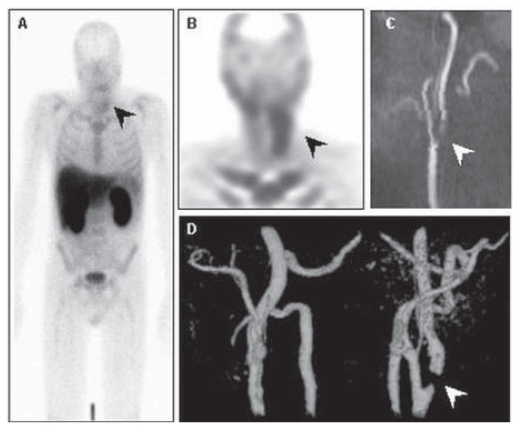
Planar (**A**) and Annexin V SPECT (**B**) images show pathological tracer uptake in the left side of the neck (black arrows), corresponding with prominent stenosis of the left carotid artery (white arrows) in the sagital (**C**) and frontal (**D**) MIP from MR angiography.

Annexin V accumulation in atherosclerotic plaques is suggested to represent plaque instability and may be used to identify patients at risk for acute vascular events^29^. Apoptosis of smooth-muscle cells and of macrophages in the plaque is thought to cause this tracer accumulation. This phenomenon is responsible for another pitfall of Annexin V interpretation in oncology, especially in patients with head and neck tumours. As illustrated in Figure 5, the radiofarmacon accumulation in the unstable plaque in carotid artery could mimic lymph node involvement.

#### Haemangioma

**Figure 6:**
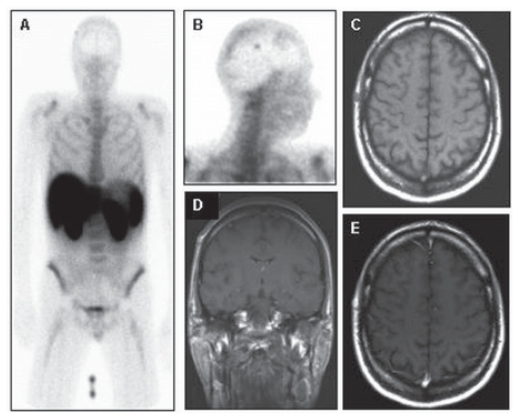
Planar anterior (**A**) and lateral (**B**) Annexin V images show focal tracer uptake in the skull right. This lesion correlates with an oval subcutaneous mass with intermediate signal intensity on T1 weighted MR image and homogeneous enhancement on coronal (**D**) and axial gadolinium-enhanced T1-weighted MR images, identified as a capillary hemangioma.

Haemangioma is a common benign vascular neoplasm that closely resembles normal vessels and that can be found in all organs of the human body. The mechanism of the Annexin V accumulation in haemangioma is not completely clear yet, however it is suggested that spontaneous apoptosis in the endothelial cells, reflecting involuntary changes in the lesion, could be responsibly for this phenomenon as illustrated in Figure 6.

### Annexin V scan interpretation after chemotherapy or radiotherapy

**Figure 7:**
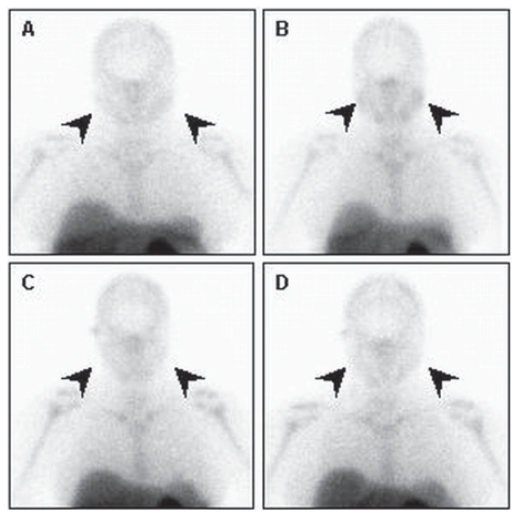
Baseline (**A**) and early post-treatment (**B**) planar Annexin V images show radiotherapy-induced increase of the tracer uptake in salivary glands (black arrows).One year later both baseline (**C**) and post-radiation (**D**) scans show no significant changes in tracer accumulation in the salivary glands (black arrows).

**Figure 8:**
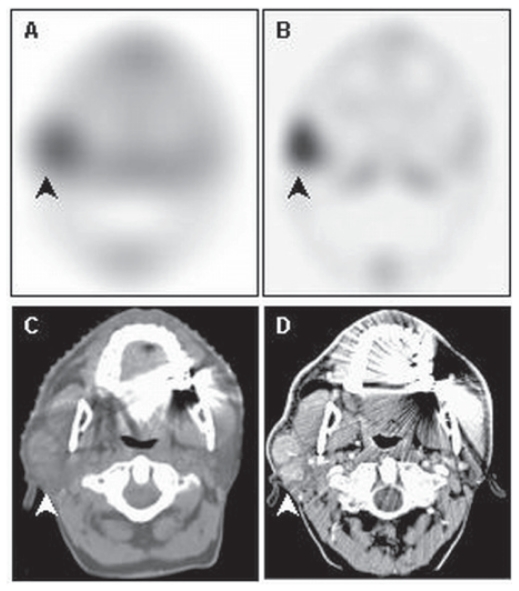
Baseline (**A**) and early post-radiotherapy (**B**) axial SPECT shows a prominent diffuse increase of tracer uptake in the right parotid gland. This change in uptake hampers the evaluation of tracer uptake in the low grade lymphoma of the left parotid gland as presented in the axial CT scan with (**D**) and without (**C**) contrast enhancement.

Anticancer treatment causes prominent changes in tumour tracer accumulation and affects tracer distribution in normal tissues. Both early and late therapy induced changes could influence tracer distribution in normal tissues. The most prominent changes occur in salivary glands and bone marrow. As reported in our previous publication, salivary glands show intense increase of tracer accumulation confined to the radiation field. It could represent radiation-induced apoptosis in the serous gland cells as demonstrated by Stephens et al 30. Tracer uptake may carry prognostic information for the early evaluation of the salivary gland damage and function. Annexin V scans performed in patients with a history of salivary gland irradiation showed minor tracer accumulation in the irradiated glands without any changes of uptake after the radiotherapy as shown in Figure 7. These findings probably illustrate irreversible radiotherapy-induced damage of the serous gland cells and may be a useful biomarker of radiation-induced salivary dysfunction. Early radiotherapy induced tracer uptake compromises tumour uptake evaluation in head and neck area, as illustrated on Figure 8.

Chemotherapy and radiotherapy-induced bone marrow tracer accumulation could reflect intramedullary apoptosis of haematopoietic tissue. As shown by Blankenberg et al, increase of Annexin V uptake in the bone marrow shortly after the start of chemotherapy, correlated with decrease in marrow cellularity affecting white and red blood cell nuclei with increasing atypia and nuclear fragmentation (apoptosis)^31^. These findings could explain diffuse increase of the tracer accumulation in the bone marrow of patients, receiving cisplatin and/or radiotherapy.

**Figure 9:**
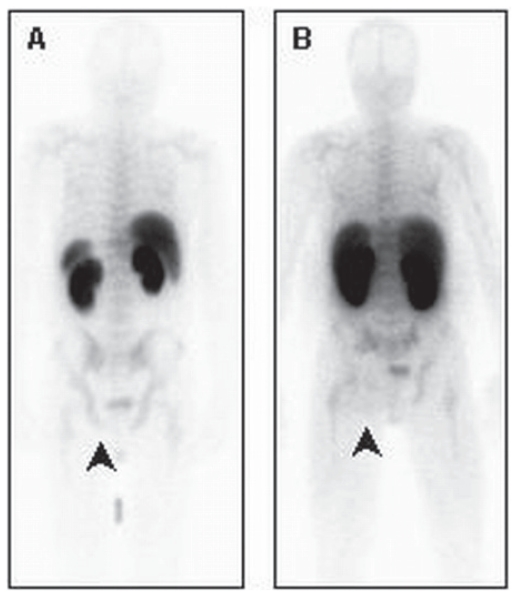
Posterior planar Annexin V images show normal (**A**) tracer uptake in the bone marrow, note diffuse tracer distribution in the pelvis. Note photopenic area (black arrow) in the projection of the left ischium on posterior Annexin V planar image (**B**). This defect corresponds with the irradiated area 1 year earlier.

Absence of tracer uptake in previously irradiated bone marrow could, thus be related to different radiosensitivity and (Figure 9) regrowth potential of the stromal and haemopoietical components with rapid regeneration of stromal tissue and replacement of the haemopoetical cells by adipocytes.

### Summary

Sequential evaluation of early therapy-induced apoptosis is a promising method to predict tumour response within 48 hours after the start of treatment in various malignant tumours. Therapy-induced increase of the tracer uptake correlates well with short-term outcome, decrease or stable tracer uptake is characteristic for unfavourable prognosis. Intensity and time-window for apoptosis imaging varies from tumour type and therapy regimen. Moreover, tumour uptake evaluation could be compromised by tracer accumulation in normal structures and non-malignant lesions. Knowledge of pattern of tracer uptake and recognition of the pitfalls of Annexin V-based apoptosis imaging could improve accurate identification and interpretation of tracer uptake to allow patient-tailored therapy.
